# Genocide Exposure and Subsequent Suicide Risk: A Population-Based Study

**DOI:** 10.1371/journal.pone.0149524

**Published:** 2016-02-22

**Authors:** Stephen Z. Levine, Itzhak Levav, Rinat Yoffe, Yifat Becher, Inna Pugachova

**Affiliations:** 1 Department of Community Mental Health, Faculty of Social Welfare and Health Sciences, University of Haifa, Haifa, Israel; 2 Department of Information and Evaluation, Ministry of Health, Jerusalem, Israel; University of Vienna, School of Psychology, AUSTRIA

## Abstract

The association between periods of genocide-related exposures and suicide risk remains unknown. Our study tests that association using a national population-based study design. The source population comprised of all persons born during1922-1945 in Nazi-occupied or dominated European nations, that immigrated to Israel by 1965, were identified in the Population Register (N = 220,665), and followed up for suicide to 2014, totaling 16,953,602 person-years. The population was disaggregated to compare a trauma gradient among groups that immigrated before (indirect, n = 20,612, 9%); during (partial direct, n = 17,037, 8%); or after (full direct, n = 183,016, 83%) exposure to the Nazi era. Also, the direct exposure groups were examined regarding pre- or post-natal exposure periods. Cox regression models were used to compute Hazard Ratios (HR) of suicide risk to compare the exposure groups, adjusting for confounding by gender, residential SES and history of psychiatric hospitalization. In the total population, only the partial direct exposure subgroup was at greater risk compared to the indirect exposure group (HR = 1.73, 95% CI, 1.10, 2.73; P < .05). That effect replicated in six sensitivity analyses. In addition, sensitivity analyses showed that exposure at ages 13 plus among females, and follow-up by years since immigration were associated with a greater risk; whereas in utero exposure among persons with no psychiatric hospitalization and early postnatal exposure among males were at a reduced risk. Tentative mechanisms impute biopsychosocial vulnerability and natural selection during early critical periods among males, and feelings of guilt and entrapment or defeat among females.

## Introduction

Protracted and severe anti-Semitic persecutions before, during [1–3] and after the Holocaust [4], termed genocide [5], generated maximum adversity among European Jews. However, documentation regarding survivor suicide risk using non-select population-based data is unavailable. Yet, theory [6], etiology [7], research regarding critical periods [8] and survivors’ morbidity [9], raise untested hypotheses.

We submit three hypotheses regarding the association between Holocaust exposure and suicide risk. Testing long-term suicide risk is consistent with clinical [7] findings that Holocaust survivors present more posttraumatic stress symptoms almost 70 years after the maximal adversities. Although those findings did not replicate in the community, survivors present more sleep problems than a suitable comparison group, likely lingering effects of traumatic events [8].

Hypothesis I proposes that Holocaust survivors emerged from adversity as a resilient group [10] with a reduced suicide risk. This hypothesis suggests that vulnerable persons characterized by suicide risk factors, such as male gender [11], and those affected by psychiatric disorders [12], may have perished during the Holocaust [i.e., selective mortality; 13]. Among these environmental factors was the systematic murder or sterilization of between 73% and 100% of persons with severe mental disorders [13]. Indeed, a community study has shown that compared to suitable controls survivors present an all-cause mortality reduction [9]. Also, among elderly persons with cancer, compared to a suitable reference group Holocaust survivors did not significantly differ on suicide risk [14]. However, that study examines an at-risk group, since it has been shown that Holocaust exposure elevates the risk of cancer [15], and old age increases the risk of suicide. In contrast, Hypothesis II proposes that exposure to multiple environmental Holocaust adversities made survivors vulnerable to psychiatric morbidity [16,17], and, possibly, suicide. For instance, prior research has shown that Holocaust exposure modestly but significantly elevates the risk of developing schizophrenia [18]. Specifically, Holocaust exposure per se, as well as combined in utero and postnatal, and late postnatal (over age 2) initiated Holocaust exposures modestly increase the risk of schizophrenia. Accordingly, an appropriate test of hypotheses I and II with regard to suicide risk is pending.

Hypothesis III submits that legal and illegal refugees who fled during the Nazi domination were at risk of suicide since they suffered directly from severe psychosocial stressors (e.g., direct persecution, bereavement) that endured during and after the Holocaust (e.g., learning that their significant others perished during the genocide). These adversities may have induced complex processes (e.g., complicated bereavement; [19]) that generated vulnerability to suicide risk [6]. Despite that, no study has empirically examined this group.

We submit three further hypotheses regarding critical biopsychosocial developmental periods of exposure to multiple adversities. These critical periods have been tested in other Holocaust research with regard to schizophrenia [10, 11], and subsequently theoretically considered by others [12]. Also, research has shown that young survivors of Nazi persecution exposed to traumatic stress, show evidence of early onset of differential neuroendocrine trajectories (notably cortical reactivity; [20,21]) indicative of stress-regulating mechanisms [22].

Hypothesis IV considers the effects of malnutrition and stress in utero, based on observations of survivor malnutrition and famine [13, 14]. For instance, the fetal origins hypothesis states that reduced fetal growth is associated with later chronic conditions [15]. This is supported by research showing that low birth weight, a marker of fetal malnutrition, is predictive of later suicide risk [1, 15]. Accordingly, in utero exposure to the maximal adversities of the Holocaust may have constituted a critical period that elevated suicide risk. Hypothesis V submits that early postnatal life (age 1–2) is another critical period. This hypothesis is based on the American Academy of Pediatrics [16] expert opinion document that states early life biopsychosocial disruptions result from cumulative damage over time or by biological embedding of adversities in critical developmental periods.

Hypothesis VI proposes that childhood (over age 2; late postnatal life) constitutes a critical period for suicide risk. This is supported by observations that severe emotional adversities (e.g., parental death at the age of seven) elevate later suicide risk [1]. Indeed, that period has been shown to be critical period for schizophrenia in other Holocaust research [10, 11]. Theoretically, childhood (ages 3–12) is a critical period based on the early-period neurodevelopmental hypothesis [17] that postulates that pre- or peri- natal etiological factors create a static neurodevelopmental deficit that remains relatively dormant. Also, sense of coherence, the primary concept in Antonovsky's salutogenic model [2], has been shown to moderate the effects of childhood Holocaust exposure among adults [3]. Lastly, hypothesis VII extends the argument of hypothesis VI to adolescence (age 13 and above), period in which the risk of suicide starts to mount [18]. This hypothesis is based on the late-period neurodevelopmental hypothesis [19] that states pathological brain maturation processes occur in adolescence.

These hypotheses were tested by contrasting genocide exposed groups of survivors with an ethnically identical comparison group with relational trauma [23] using a population-based study design.

## Materials and Methods

This study received ethical approval from the Institutional Review Board (IRB) at the University of Haifa, Israel. That IRB waived the requirement of written informed consent and approved linking the study data sources, since no identifiable personal information was made available to the authors. Common unique identification numbers were encrypted prior to receiving the data to ensure participant anonymity and confidentiality. The Ministry of the Interior facilitated the identification of the study population and causes of death; the Central Bureau of Statistics provided the socioeconomic status information; and the Ministry of Health facilitated the psychiatric hospitalization information.

*Nation-wide Population Register of the Ministry of Interior*. The study population comprised of all Jews born in Holocaust-exposed European countries from 1922 to 1945, who immigrated to Israel by 1966. These birth cohorts were chosen since approximately 90% of Holocaust survivors who immigrated to Israel were born after 1920. The study data were at the individual-level and comprised of encrypted identification numbers, nation of origin, years of birth, immigration, residential area code, death year, causes of death.

Suicide ascertainment: Records from the Ministry of the Interior provided information on causes of death, including suicide. The causes of death are recorded according to the ICD-10 codes, and updated for past ICD codes. The entry of suicide is made by the Central Bureau of Statistics with information provided by the regional Ministry of Health offices and certified by the National Forensic Institute when the police delivered the body for autopsy.

*Central Bureau of Statistics (CBS) Registry*. This registry enabled the identification of each person’s residential status area (i.e., a neighborhood measure of socioeconomic status; SES). This measure is derived from household census data that are based on number of electrical appliances per person and per capita income [24]. Residential status was obtained by linking each individual address from the Population Register of the Ministry of Interior with the CBS census track and neighborhood ranking.

*National Psychiatric Case Registry (NPCR)*. Psychiatric care in Israel is freely available by law to all de-jure residents. The law mandates that all inpatient and day care psychiatric settings submit information on all admissions to the Ministry of Health that verifies reporting compliance, updating diagnostic coding schemes and information consistency. The information received from the registry included admission and discharge dates, and ICD-10 discharge and admission diagnoses made by a board–certified psychiatrist. The current study used the last registry diagnosis at the time of discharge, or upon admission if the hospitalization was current or the patient died in hospital. The clinical diagnosis in the NPCR registry have acceptable sensitivity and specificity when assessed against research diagnosis [25], and is longitudinally reliable [26].

### Analytical strategy

Exposure groups and subgroups. The population studied was disaggregated into exposure groups and subgroups. All persons in the population had national differences accounted for by the periods that the Nazi domination began [for the year that Nazi domination began in each nation see: 18].

Across analyses, the comparison group comprised of Jewish persons born in European nations where the Holocaust occurred and that immigrated to pre-State Israel before the anti-Semitic persecutions began or markedly increased. This group did not directly experience the Holocaust, but likely had family and social ties (relational trauma) to people who did. [23]. This group is identified here as likely “*indirect”* exposure group.

Periods of Holocaust exposure were used to classify two groups. The likely direct “*partial”* exposure group was defined here as all persons in the study population that immigrated a) after the persecutions began in their nation of origin [for the year that Nazi domination began in each nation see: 18]; and b) arrived in Israel before World War II ended. This group of refugees probably directly witnessed or suffered Nazi persecutions in their nation of origin, and later sought shelter by fleeing to pre-State Israel. The likely “*full direct*” exposure group comprised of all persons in the study population that immigrated in Israel after the end of World War II and had gone through it in Europe.

To test the hypothesis that exposure to critical developmental periods of multiple adversities elevate suicide risk, the likely *partial and full* direct exposure groups were further classified into subgroups. These subgroups were: (a) “*Likely in utero only*”, if persons were born in the last year of the war (i.e., 1945) and hence, unlike the remaining groups, comprised of full exposure only; (b) “*Likely in utero and postnatal*” subgroup, if they were born during the Nazi domination and survived a period of postnatal exposure (e.g., German births during 1933–44); and (c) “*Likely early postnatal*” subgroup, if the initial Holocaust exposure occurred by ages 1–2 (e.g., if born in Germany in 1931–1932), that coincides with the critical period of development up to the age of two, as defined by an expert consensus [27]; and “*Likely late postnatal*” subgroup (e.g., Germany born: 1922–1931). To better identify critical periods of late postnatal development, the subgroup was further disaggregated into initial exposure subgroups of (d) “*Likely at ages 3–12*” and (e) “*Likely 13 and above*” in the analysis.

### Statistical analysis

First, descriptive statistics, and suicide numbers, percentages and incidence rates per 100,000 person-years of completed suicide were calculated. Rates were computed using a previously validated and applied function in the R programming language [28]. Second, to examine the primary study outcome of age at suicide, survival analyses were computed with Kaplan-Meier and Cox regression modeling. Kaplan-Meier estimates were computed and plotted to examine the average age of suicide all groups. Hazard Ratios (HR) were computed from Cox regression as the primary analysis to test the study hypotheses of subgroup differences applying standard statistical guidelines [29]. The HR expresses the ratio between groups in instantaneous suicide risk over the study time. It may be thought of as the expression of the chance of events occurring in the exposure group as a ratio of the hazard of the events occurring in the indirect exposure group. HR values where the associated 95% confidence intervals crosses the unity indicates a not statistically significant result. Since in our analytical models, the indirect exposure group was used as the reference group, a Hazard Ratio and corresponding 95% confidence intervals over 1 indicate a greater suicide risk for the exposed subgroup. Often, an HR value of up to 1.3 is thought to reflect a small effect size, up to 1.5 a medium effect size, and over 2.0 a large effect size [30]. The survival times were computed to account for censoring (i.e., death of subject, or end of follow-up without suicide completion). Using the survival package in R software [31], Cox models were computed adjusting for confounding by gender, residential SES and presence of a psychiatric hospitalization.

Sensitivity analyses were recomputed for the aforementioned primary analysis four times, each time adjusting for a different potential confounder. First, the primary outcome variable was changed from age to year since immigration to account for the beginning of the follow-up time. Second, persons hospitalized for any psychiatric disorder were removed from the analysis. They were removed since Holocaust exposure modestly yet significantly elevated the risk of psychiatric hospitalization for schizophrenia in these data [18] and elsewhere psychiatric hospitalization is documented risk factor for suicide [32]. Third, sensitivity analyses were computed by gender since generally, men are at greater suicide risk than women [33]. However, possible interactions between gender and Holocaust exposure on suicide risk have not been examined. The fourth sensitivity analysis was computed to account for famine by removing persons born in the Ukraine (n = 3605, suicides: 5) because a famine occurred there 1932–33 known as the *Holodomo*r. The fifth sensitivity analysis was computed retaining persons born in Greece only, as famine occurred there from 1941–1944; as well as in Poland, where famine occurred from 1940–1945. Sixth, data were reanalyzed for all persons in the population who were Poland-born. This group is particularly relevant as the Nazis inflicted famine in camps and many ghettos (1940–1945). This subgroup was most extensively exposed to the Holocaust; approximately 90% of Poland-born Jews were murdered [4,34]. The 10% of Polish-born Jews that survived constituted the largest post-WWII survivor community to migrate to Israel. These survivors were reported to have had a lower all-cause mortality by six months than similar migrants prior to WWII [9].

## Results

The study population (N = 220,665) was comprised of more females (n = 115,905, 52.5%) than males (n = 104,760, 47.5%), and predominantly by the full direct exposure subgroup (82.9%; Table 1). ANCOVA to examine birth year by likely exposure group showed a significant main effect (F = 4337.976, df = 220662, 2, P < .05). The full direct group was born approximately two years later than the remaining groups (indirect M = 1927.16, 95% CI 1927.11, 1927.22; partial M = 1927.32, 95% CI 1927.25, 1927.39; full direct M = 1929.94, 95% CI 1929.92, 1929.97). ANCOVA to examine follow up time by likely exposure group showed a significant main effect (F = 475.99.99, df = 220662, 2, P < .05). The full direct group had approximately two years significantly fewer years of follow up than the remaining groups (indirect M = 78.46, 95% CI 78.30, 78.63; partial M = 78.61, 95% CI 78.42, 78.79; full direct M = 76.48, 95% CI 76.43, 76.53).

**Table 1:** Maximal adversity exposure groups and the risk of suicide.

Model approach:	Suicide numbers		Follow-up per100,000 person-years	Rate per 100,000 person-years		Kaplan-Meier		Adjusted Cox	
**Likely exposure**	**Absent (N)**	**Present (N)**	**Person-years**	**Rate**	**(95% CI)**	**M**	**(95% CI)**	**HR**^1^	**(95% CI)**
**Indirect**	20580	32	1617267	1.98	(1.292.66)	91.94	(91.92, 91.96)	Ref.^2^	
**Partial**	16992	45	1339247	3.36	(2.38 4.34)	91.91	(91.88, 91.94)	**1.73**	(1.10, 2.73)
**Direct**	182762	254	13997088	1.81	(1.592.04)	91.95	(91.94, 91.96)	0.92	(0.64, 1.34)
**Likely exposure**	**Absent (N)**	**Present (N)**	**Person-years**	**Rate**	**(95% CI)**	**M**	**(95% CI)**	**HR**^1^	**(95% CI)**
**Indirect**	20580	32	1617267	1.98	(1.292.66)	91.94	(91.92, 91.96)	Ref. ^2^	
**In utero only**	5819	2	389611	0.51	-(0.201.22)	68.99	(68.97, 69.00)	0.25	(0.06, 1.06)
**In utero & postnatal**	23391	24	1649211	1.46	(0.872.04)	79.97	(79.95, 79.98)	0.70	(0.41, 1.19)
**Early postnatal**	12800	10	940399	1.06	(0.401.72)	81.98	(81.96, 81.99)	0.52	(0.25, 1.05)
**Late postnatal**	157744	263	12357114	2.13	(1.872.39)	91.94	(91.94, 91.95)	1.10	(0.76, 1.59)
**Likely exposure**	**Absent (N)**	**Present (N)**	**Person-years**	**Rate**	**(95% CI)**	**M**	**(95% CI)**	**HR**^1^	**(95% CI)**
**Indirect**	20580	32	1617267	1.98	(1.292.66)	91.94	(91.92, 91.96)	Ref. ^2^	
**In utero only**	5819	2	389611	0.51	-(0.201.22)	68.99	(68.97, 69.00)	0.25	(0.06, 1.06)
**In utero & postnatal**	23391	24	1649211	1.46	(0.872.04)	79.97	(79.95, 79.98)	0.70	(0.41, 1.19)
**Early postnatal**	12800	10	940399	1.06	(0.401.72)	81.98	(81.96, 81.99)	0.52	(0.25, 1.05)
**3 to 12**	95264	145	7370171	1.97	(1.652.29)	91.94	(91.94, 91.95)	1.00	(0.68, 1.47)
**13 plus**	62480	118	4986943	2.37	(1.942.79)	91.94	(91.93,91.95)	1.25	(0.84, 1.85)

^1^ HR is the Hazard Ratio, referring to the risk over the study time with statistically significant (P < .05) values in bold, adjusted for sex, socioeconomic status and psychiatric hospitalization groups. An HR value of up to 1.3 is often interpreted as a small effect size, up to 1.5 a medium effect size, and over 2.0 a large effect size [30].

^2^ Ref. refers to the reference group

**Fig 1:**
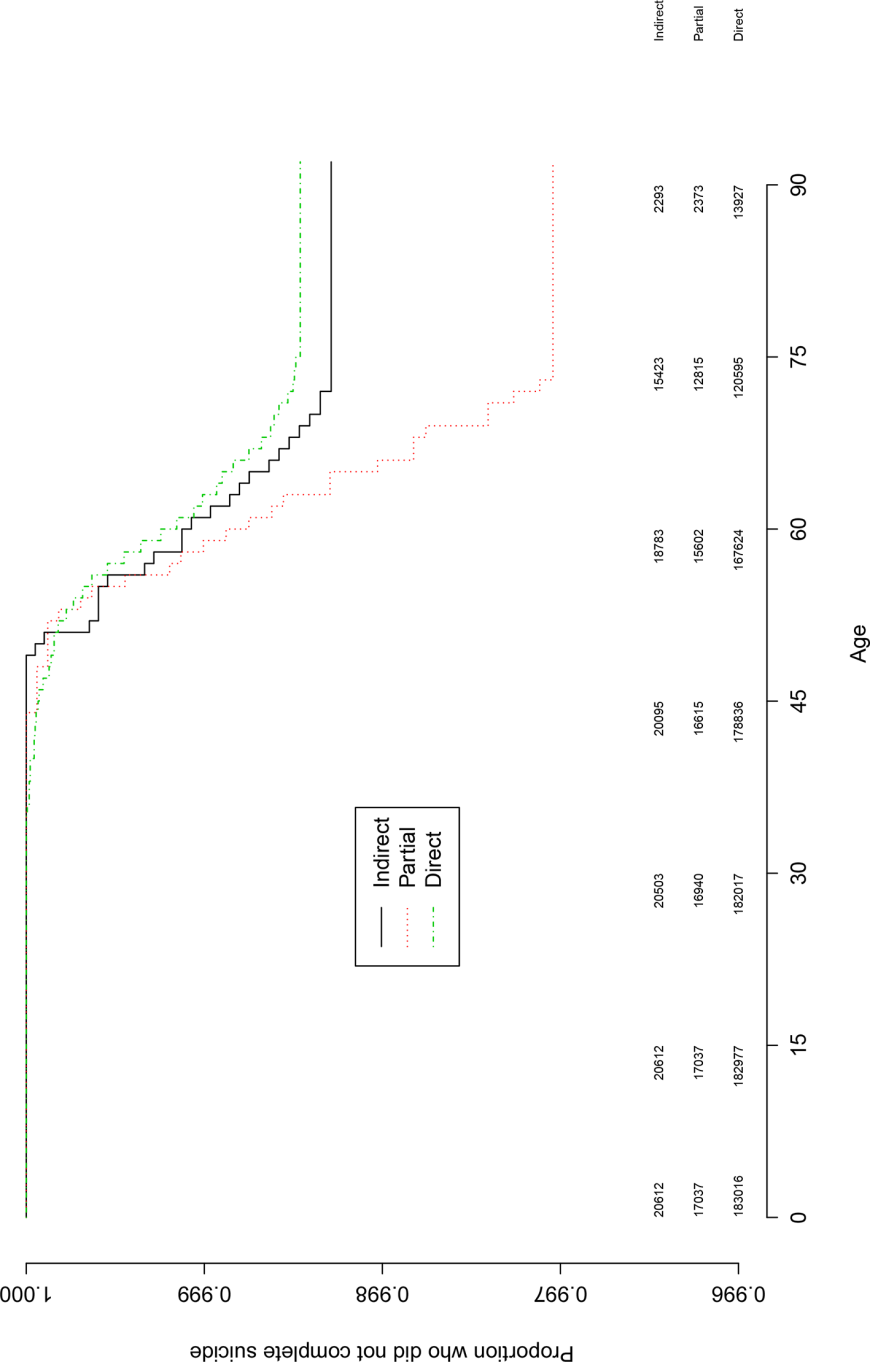
Kaplan-Meier survival curve for the cumulative probability of suicide risk for the indirect, partial and direct exposure groups. Note. The curves depict average age of suicide for the exposure groups based on Kaplan-Meier modeling estimates. The vertical axis is the proportion that did not complete suicide. The numbers under the horizontal axis are age, and the numbers above the x-axis are the numbers at risk every 15 years.

**Fig 2:**
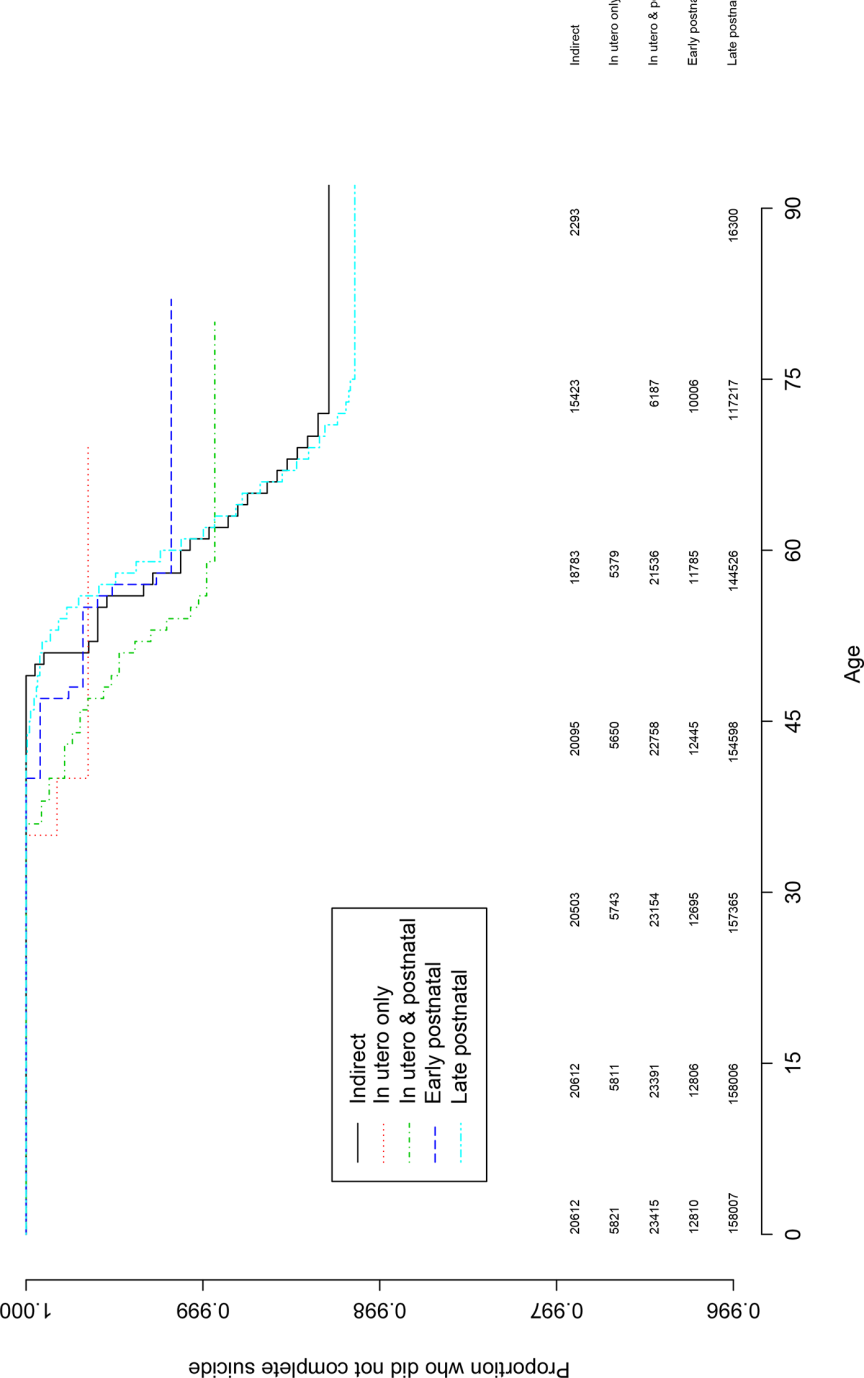
Kaplan-Meier survival curve for the cumulative probability of suicide risk for the indirect, in utero only, in utero and postnatal and late postnatal exposure subgroups. Note. The curves depict average age of suicide for the exposure groups based on Kaplan-Meier modeling estimates. The vertical axis is the proportion that did not complete suicide. The numbers under the horizontal axis are age, and the numbers above the x-axis are the numbers at risk every 15 years.

**Fig 3:**
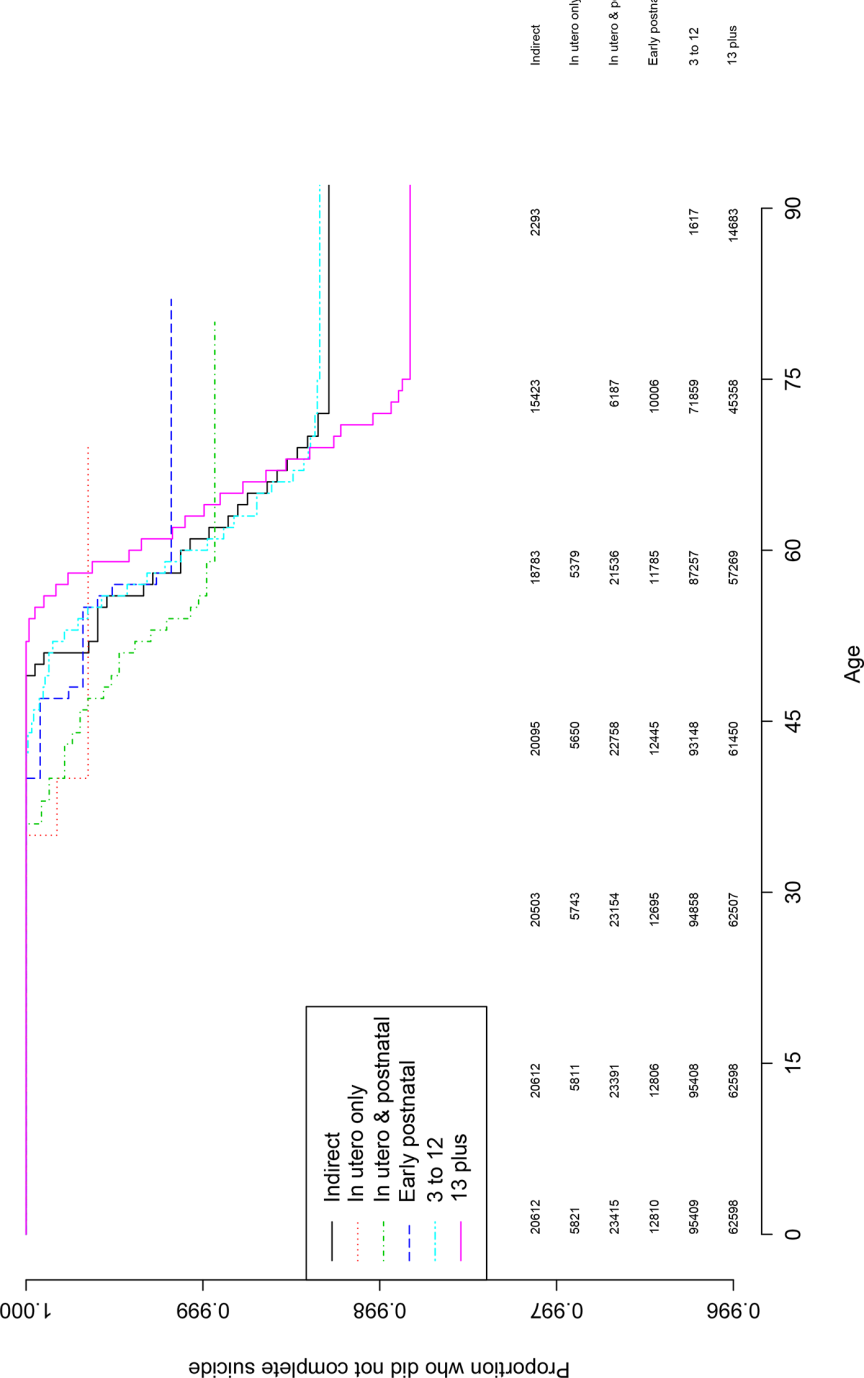
Kaplan-Meier survival curve for the cumulative probability of suicide risk for the indirect, in utero only, in utero and postnatal exposure, ages 3 to 12 and 13 plus exposure subgroups. Note. The curves depict average age of suicide for the exposure groups based on Kaplan-Meier modeling estimates. The vertical axis is the proportion that did not complete suicide. The numbers under the horizontal axis are age, and the numbers above the x-axis are the numbers at risk every 15 years.

The full direct exposure group had the lowest suicide rate, and in a descending order, the indirect and the partial exposure groups (see Table 1). The exposure groups were compared on the risk of suicide using three Cox regression models. Compared to the likely indirect exposure group, the partial direct exposure subgroup was at a statistically significant increased suicide risk with a moderate effect size (HR = 1.73, 95% CI 1.10, 2.73; P < .05; Table 1). Compared to the indirect exposure subgroup, the remaining direct exposure subgroups did not significantly differ (Table 1). That is illustrated in Figs 1, 2 and 3 with the numbers at risk every 15 years.

### Sensitivity analyses

Sensitivity analyses comprised of re-computing the primary analysis in six different ways accounting for censoring appropriately (Table 2). First, the number of years from immigration to suicide was computed. The analysis replicated the above result: only the partial direct exposure subgroup was at a statistically significant increased risk of suicide with a moderate effect size (HR = 1.81; 95% CI, 1.15, 2.85; P < .05) compared to the indirect exposure group. Also, only the initial exposure of the subgroup at 13 years of age or older was at a statistically significant risk of suicide with a moderate effect size (HR = 1.58; 95% CI, 1.07, 2.34; P < .05) compared to the indirect exposure group.

**Table 2:** Sensitivity analyses.

Sensitivity analysis^2^:	Year since immigration		Persons with a psychiatric hospitalization removed		Among females only		Among males only		Ukrain-born removed		Among Poland & Greek born		Among Poland-born	
**Likely exposure group**	**HR**^1^	**(95% CI)**	**HR**^1^	**(95% CI)**	**HR**^1^	**(95% CI)**	**HR**^1^	**(95% CI)**	**HR**^1^	**(95% CI)**	**HR**^1^	**(95% CI)**	**HR**^1^	**(95% CI)**
**Partial**	**1.81**	(1.15, 2.85)	**1.62**	(1.03, 2.55)	**4.59**	(1.83, 11.51)	1.15	(0.66, 1.98)	**1.65**	(1.04, 2.61)	**2.39**	(1.07, 5.33)	**1.73**	(1.10, 2.73)
**Direct**	1.07	(0.74, 1.55)	0.91	(0.63, 1.31)	1.66	(0.72, 3.79)	0.76	(0.50, 1.14)	0.88	(0.61, 1.29)	1.34	(0.77, 2.34)	0.92	(0.64, 1.34)
**Likely exposure group**	**HR**^1^	**(95% CI)**	**HR**^1^	**(95% CI)**	**HR**^1^	**(95% CI)**	**HR**^1^	**(95% CI)**	**HR**^1^	**(95% CI)**	**HR**^1^	**(95% CI)**	**HR**^1^	**(95% CI)**
**In utero only**	0.24	(0.06, 1.02)	**0.23**	(0.06, 0.97)	0.77	(0.09, 6.43)	0.15	(0.02, 1.09)	0.24	(0.06, 1.02)	0.93	(0.12, 7.02)	0.25	(0.06, 1.06)
**In utero & postnatal**	0.73	(0.43, 1.25)	0.67	(0.39, 1.13)	0.33	(0.07, 1.64)	0.77	(0.44, 1.37)	0.68	(0.40, 1.16)	0.24	(0.03, 1.80)	0.70	(0.41, 1.19)
**Early postnatal**	0.56	(0.27, 1.13)	0.50	(0.24, 1.01)	2.35	(0.81, 6.78)	**0.12**	(0.03, 0.52)	0.50	(0.24, 1.02)	0.57	(0.13, 2.50)	0.52	(0.25, 1.05)
**Late postnatal**	1.30	(0.90, 1.88)	1.09	(0.75, 1.57)	2.07	(0.91, 4.73)	0.88	(0.58, 1.33)	1.05	(0.72, 1.53)	1.57	(0.90, 2.73)	1.10	(0.76, 1.59)
**Likely exposure group**	**HR**^1^	**(95% CI)**	**HR**^1^	**(95% CI)**	**HR**^1^	**(95% CI)**	**HR**^1^	**(95% CI)**	**HR**^1^	**(95% CI)**	**HR**^1^	**(95% CI)**	**HR**^1^	**(95% CI)**
**In utero only**	0.24	(0.06, 1.02)	**0.23**	(0.06, 0.97)	0.77	(0.09, 6.43)	0.15	(0.02, 1.09)	0.24	(0.06, 1.02)	0.93	(0.12, 7.03)	0.25	(0.06, 1.06)
**In utero & postnatal**	0.73	(0.43, 1.25)	0.67	(0.39, 1.13)	0.33	(0.07, 1.64)	0.77	(0.44, 1.37)	0.68	(0.40, 1.16)	0.24	(0.03, 1.80)	0.70	(0.41, 1.19)
**Early postnatal**	0.56	(0.27, 1.13)	0.50	(0.24, 1.01)	2.35	(0.81, 6.77)	**0.12**	(0.03, 0.52)	0.50	(0.24, 1.02)	0.57	(0.13, 2.50)	0.52	(0.25, 1.05)
**3 to 12**	1.14	(0.77, 1.67)	0.99	(0.67, 1.45)	1.84	(0.79, 4.30)	0.81	(0.52, 1.25)	0.97	(0.66, 1.43)	1.39	(0.76, 2.55)	1.00	(0.68, 1.47)
**13 plus**	**1.58**	(1.07, 2.34)	1.24	(0.84, 1.83)	**2.41**	(1.03, 5.66)	0.98	(0.62, 1.53)	1.17	(0.79, 1.75)	1.75	(0.98, 3.15)	1.25	(0.84, 1.85)

^1^ HR is the Hazard Ratio, referring to the risk over the study time with statistically significant (P < .05) values in bold. They are adjusted for sex, socioeconomic status and psychiatric hospitalization groups. An HR value of up to 1.3 is often interpreted as a small effect size, up to 1.5 a medium effect size, and over 2.0 a large effect size [30]. Covariates were not included if relevant persons were excluded from sensitivity analysis (e.g., sex in the analysis of females).

^2^ The reference group is the likely indirect exposure group.

Second, the primary analysis was re-computed removing persons with a history of psychiatric hospitalization [18]. The analysis replicated the result of the primary analysis: the partial direct exposure subgroup was at a statistically significant higher risk of suicide with a moderate effect size (HR = 1.62; 95% CI, 1.03, 2.55; P < .05) compared to the indirect exposure group (Table 2). Also, the in utero only exposure subgroup was at a reduced suicide risk (HR = 0.23, 95% CI, 0.06, 0.97; P < .05; Table 2) compared to the indirect exposure group (Table 2).

Third, for each possible exposure—gender interaction, a Cox regression model was computed. Results showed statistically significant interactions for each exposure (see S1 Table). To better understand the form of the interaction, the primary analysis was re-computed stratifying for each gender. Among females, only the partial exposure subgroup (HR = 4.59; 95% CI, 1.83, 11.51; P < .05), and the subgroup exposed at age 13 and over (HR = 2.41; 95% CI, 1.03, 5.66; P < .05) were at a significant suicide risk with strong effect size magnitudes compared to the indirect exposure group (see Table 2). Among males, compared to the indirect exposure group, the *early postnatal* subgroup was at reduced risk (HR = 0.12; 95% CI, 0.03, 0.52). All remaining group contrasts were not statistically significant (see Table 2).

Fourth, after removing all persons born in the Ukraine, the likely partial exposure group was at significant suicide risk of medium effect size (HR = 1.65, 1.04, 2.61). Fifth, retaining Poland and Greece—born persons only, the likely partial exposure group was at a significant risk of suicide (HR = 2.39, 1.07, 5.33) with a strong effect size. Sixth, retaining Poland-born persons only, the likely partial exposure group was at significant suicide risk, (HR = 1.73, 1.10, 2.73) with a medium effect size. The remaining results were not statistically significant.

A post-hoc analysis was computed because the group of persons that completed suicide with in utero exposure was small, as often occurs in suicide studies [35]. Post-hoc analysis replicated the results by dropping the likely *in utero* group from analysis. Like the primary analysis, only the partial exposure group was at significant suicide risk with a moderate effect size (HR = 1.73; 95% CI, 1.10, 2.73, P<0.05). This indicated that the inclusion of the likely *in utero* group did not compromise the study results.

## Discussion

Population-based data were followed-up to age 90 to examine six hypotheses of genocide- exposure and completed suicide. Partially supporting the study hypotheses, the primary results show that exposure within a select historical and biopsychosocial critical periods elevated the risk of suicide. Interestingly, the sensitivity analyses showed elevated suicide risks for women and reduced risks for men at different periods.

Hypothesis I, selective mortality left alive only resilient Holocaust survivors at reduced suicide risk, received some support. Specifically, in sensitivity and not in the primary analysis a reduced risk of suicide was observed among: a) males with initial exposure at ages 1–2; and b) among persons without a psychiatric hospitalization in the *in utero* only exposure subgroup [9,16]. Our findings, that early postnatal life among males is a critical period, converge with the critical period up to the age of two [27] and indicates an interaction of mechanisms comprised of resilience and natural selection processes at this age. A possible mechanism to explain the risk reduction of the *in utero* only exposure group among persons not hospitalized is alluded to in Chinese famine research [36]. That implies greater natural selection and biological resilience of early postnatal exposed males. This may be due to epigenetic modifications. These are heritable, but reversible, regulation of gene expression mediated principally through changes in DNA methylation and chromatin structure due to environmental mechanisms [37]. These epigenetic modifications themselves may explain how trauma, caused by environmental exposure, modulates gene expression without necessarily impacting on the genetic code [38].

The finding that the full direct and indirect exposure groups did not differ in suicide risk is consistent with other studies showing unusual resilience among survivors (Hypothesis II). For instance, Poland-born Holocaust survivors lived 6 months longer than Poland-born Israelis who immigrated before WWII [9]. Also, among elderly persons with cancer, a select group at high-risk for suicide, Holocaust survivors were at equal risk to a comparable population group [14].

Hypothesis II, exposure to maximum adversities induced multiple vulnerabilities, was partly supported. Results showed that women with initial exposure at age 13 years and onward were at risk of subsequent suicide in specificity analysis only. Possible mechanisms involved may be genes (e.g., serotonin transporter 5-HT T) and/or bereavement interacting with traumatization [39,40].

Hypothesis III was supported in women. That suggests that experiencing and fleeing severe persecution while significant others suffered and/or perished, induced complicated late reactions of bereavement [19], guilt and/or entrapment and defeat thus increased the risk of suicide [6]. This result is consistent with findings from a Scandinavian study that demonstrated that compared to fathers, mothers were at excess risk of early unnatural mortality (including suicide) following the death of a child [19,41]. Tentative mechanisms involved in the elevation of female suicide risk include guilt and entrapment or defeat [6], and/or female empathy [42].

Hypothesis IV, contrary to the early life hypotheses [7,43], we observed no elevated risk due to *in utero* exposures. Rather reduced suicide risk was observed among persons without a psychiatric hospitalization in the *in utero* only exposure group, and among males in the *early postnatal* (age 1–2) exposure group. The aforementioned possible mechanisms aim to explain those findings comprise of greater biological resilience, and hence greater natural selection during exposure at those critical periods [36]. Contrary to hypothesis V, childhood Holocaust exposure was not significantly associated with suicide [8]. Unlike childhood exposure, however, as stated by hypothesis VI, exposure during adolescence elevated suicide risk, was supported among females with initial exposure at 13 years of age or older. Mechanisms responsible for this suicide risk trajectory may include guilt and entrapment or defeat [6], and/or female empathy [42].

By design, the study reference group had likely relational trauma [23].This raised the stringency of the comparator group by reducing the difference with the direct and indirect exposure groups. Of interest, however, is to consider this study suicide rates to existing rates in the literature. The latest Israeli government suicide report states that between 2006 and 2011 peak rates in the population were at aged 75 or over with a 32.9 per 100,000 rate in the population, and among males, at 45.6, and females, at 22.0 both per 100,000 in the population [44]. Another comparison may be to consider Israeli immigrants from the former Soviet Union. They constitute 17% of the population yet contribute up to 26% of completed suicides. However, they are of mixed ethnic origin, and that makes any comparison difficult [45]. Among these males, the suicide rate is 25.2 per 100,000 in the population. From the age 45–64, males in this group have a suicide rate of 18.9, and females, of 5.5 per 100,000. These and other available existing Israeli-estimates do not account for follow-up times, and do not examine birth cohorts. In contrast, the present study computed person-year rates that account for an extensive life-time follow-up (1950–2014 with a maximum age of 92), and provided rates for a priori defined birth cohorts (born 1922–1945). In comparison to other reported Israeli suicide rates, the raw rates of our sample are higher in the indirect (i.e. likely relational trauma; 0.16%), partial (0.26%) and direct exposure groups (0.14%). These differences between the rates may be attributable to: the effects of genocide exposure in our study population, an extensive follow-up period unavailable in any other data source with genocide exposure, and may suggest that even familial exposure to suicide had adverse consequences.

To examine famine we analyzed the subgroup born in Poland and Greece collectively. We were, however, unable to analyze a single country beyond Poland, because of the rarity of suicide. It is likely, however, that not only in camps but other nations suffered from famine in some areas only (e.g., the Netherlands). However, the inclusion of the Netherlands in our data would have resulted in misclassification, because we had information by nation and not by area. Nonetheless, during WWII it is notable that 20 million people perished from malnutrition and its associated diseases or starved to death [46]. Furthermore, inhumane Nazi food policies implemented in camps and the civilian population, that we lacked data on, included extreme food deprivation frequently resulting in death even after WWII [47]. Hence, although we did not examine famine per-se, it is likely that we ecologically captured famine-exposed groups with reasonable ecological validity because Greece and Poland were both exposed to hunger and malnutrition famine. Indeed, the results of those analyses replicated the results of the primary analysis that direct partial exposure elevated the risk of suicide.

### Limitations and conclusions

There are at least five limitations to the current study. First, since 6 of the 9 million Jews of Europe were murdered by the Nazis, mortality selection effects are expected. Our results show some evidence of those effects post World-War II, particularly among males, who probably completed suicide more often than women during the Holocaust [33]. Second, the exact exposure periods are almost impossible to identify with the available databases. For instance, some babies may have been prematurely born thereby reducing the exactness of classification for the prenatal exposure group. Nonetheless, to address the timing of exposure and the fact that adversities differed by nation we accounted for the period of exposure of Nazi persecution within each nation by birth year. Hence, our approach to defining exposure periods with a conservative “likely” denomination resembles famine studies that used well-defined periods and areas to define exposure as an ecological measure [48]. Specifically, famine studies classify individuals as being with “*likely* famine exposure” based on their presence in a famine setting using the above criteria. Accordingly, current studies consider famine exposure as an ecological measure, inasmuch as there are no data on individual food intakes. Nonetheless, the likely famine exposure has been validated by its association with fertility, pregnancy weight gain, size at birth, and mortality [48]. Third, as in any other suicide registry, it is likely that suicide rates were probably underestimated. Prior reports on the National Death Registry have offered mixed findings of its reliability [49]. Also there appears to be no reason to assume that misdiagnoses were more frequent in one group or another. A possible alternative would be to conduct a psychological autopsy study, yet such a study raises several challenges. Recall bias is likely to be pronounced due to the traumatic nature of the Holocaust. Also, it is a family subject that it is shrouded in secrecy as many survivors wanted to spare their children from exposure to the many extreme hardships that they endured. That secrecy would make knowing the nature of parental adversities challenging for a psychological autopsy study. Fourth, we omitted a non-immigrating Jewish-Israeli group for comparison purposes. Such a group could have introduced mixed ethnicity, making comparison less stringent (i.e., a comparison group without relational exposure; a possible different gene pool). Additionally, non-immigrant Jews of European descent born during the study period likely had family-based relational trauma.

A fifth set of limitations refer to the types and periods of trauma exposure. The number of Holocaust victims differed across the period of Nazi domination. It is not possible to conclude based on our resources that very early exposure was worse than later exposure. However, it has been documented that the Holocaust murder rate was worst in 1942. On 20 January 1942 the Wannsee Conference convened senior Nazis and decided the “final solution of the Jewish problem” meant total annihilation. Subsequent research documents that the number of Holocaust murders peaked in 1942 when approximately 2,7 million Jews were murdered, with 1,2 million murdered pre-1942 and 1,2 million post-1942 when fewer Jews remained alive [20]. Hence it is problematic to exactly ascertain the constancy of the adversities over the Holocaust. However, it is clear that maximal adversities occurred and remained. Indeed, even post-war atrocities occurred (e.g., in Poland there were post-war pogroms; e.g., in the city of Kielce, July 1946). Furthermore, the adversities were heterogeneous [21] and survivors who had been in ghettos, hiding, or labor or extermination camps had more emotional distress in that increasing order of persecution [22]. Therefore, future research is warranted to replicate our findings with well-defined exposure assessments.

Several strengths balance the study limitations. These include, a gradient comprised of two groups with trauma exposure, with a comparison group that were likely indirectly affected by relational trauma [23]. This study design consideration raises the stringency of the comparator group inasmuch as it reduces the difference with the direct exposure groups. Also, the study design was strengthened by the use of documented psychiatric admissions, a national population-based case registry that captures most individuals with schizophrenia [50], a previously validated death registry, a large sample size, and an extensive follow-up period.

In conclusion, the current study results demonstrate that compared to suitable counterparts, survivors of the Holocaust were not at elevated risk of suicide. Notably, this study, that is unique inasmuch as it examined refugees, found that female refugees who fled Europe while the Holocaust were at an increased suicide risk. That suggests that female refugees who flee amidst genocide constitute a special group for preventive suicide measures. Additionally, women initially exposed in adolescence were at elevated risk of suicide, whereas men exposed in utero were at reduced risk. In contrast, tentative mechanisms of biological resilience and natural selection left males with *in utero* exposure resilient with regard to suicide.

## Supporting Information

S1 Table Cox regression model terms of the sex by likely exposure group interactions. Note. Female sex and indirect exposure were reference groups.
(DOCX)
