# Supplementary material for: Genocide Exposure and Subsequent Suicide Risk: A Population-Based Study
Source: PLoS One. 2016 Feb 22;11(2):e0149524. doi: 10.1371/journal.pone.0149524 (PMC4763158; doi:10.1371/journal.pone.0149524)
Supplement: S1 Table — Note. Female sex and indirect exposure were reference groups. (DOCX) [file pone.0149524.s001.docx]

**S1 Table 1. Cox regression model terms of the sex by likely exposure group interactions.**

| **Likely exposure group** | **HR^1^** | **(95% CI)** | |
| --- | --- | --- | --- |
| Sex (male) * Partial | **0.25** | (0.09 | , 0.72) |
| Sex (male) * Direct | 0.46 | (0.18 | , 1.16) |
| **Likely exposure group** | **HR^1^** | **(95% CI)** | |
| Sex (male) * In uterus only | 0.20 | (0.01 | , 3.70) |
| Sex (male) * In uterus & postnatal | 2.45 | (0.45 | , 13.36) |
| Sex (male) * Early postnatal | **0.06** | (0.01 | , 0.34) |
| Sex (male) * Late postnatal | 0.42 | (0.17 | , 1.07) |
| **Likely exposure group** | **HR^1^** | **(95% CI)** | |
| Sex (male) * In uterus only | 0.20 | (0.01 | , 3.70) |
| Sex (male) * In uterus & postnatal | 2.45 | (0.45 | , 13.36) |
| Sex (male) * Early postnatal | **0.06** | (0.01 | , 0.34) |
| Sex (male) * 3 to 12 | 0.44 | (0.17 | , 1.15) |
| Sex (male) * 13 plus | 0.40 | (0.15 | , 1.06) |

Note. Female sex and indirect exposure were the reference group. ^1^ HR is the Hazard Ratio, referring to the risk over the study time with statistically significant (P<.05) values in bold.
